# Effects of Different TiO_2_ Particle Sizes on the Microstructure and Optical Limiting Properties of TiO_2_/Reduced Graphene Oxide Nanocomposites

**DOI:** 10.3390/nano9050730

**Published:** 2019-05-11

**Authors:** Yuyu Ren, Lili Zhao, Yang Zou, Lixin Song, Ningning Dong, Jun Wang

**Affiliations:** 1 Key Laboratory of Inorganic Coating Materials CAS, Shanghai Institute of Ceramics, Chinese Academy of Sciences, 1295 Dingxi Road, Shanghai 200050, China; renyuyu@student.sic.ac.cn (Y.R.); zouyang@mail.sic.ac.cn (Y.Z.); lxsong@mail.sic.ac.cn (L.S.); 2 University of Chinese Academy of Sciences, Beijing 100049, China; 3 Center of Materials Sciences and Optoelectronic Engineering, University of Chinese Academy of Sciences, Beijing 100049, China; n.n.dong@siom.ac.cn (N.D.); jwang@siom.ac.cn (J.W.); 4 Laboratory of Micro-Nano Optoelectronic Materials and Devices, Key Laboratory of Materials for High-Power Laser, Shanghai Institute of Optics and Fine Mechanics, Chinese Academy of Sciences, Shanghai 201800, China

**Keywords:** optical limiting, TiO_2_, reduced graphene oxide, nanocomposites

## Abstract

TiO_2_/reduced graphene oxide (rGO) nanocomposites with two different TiO_2_ particle sizes were synthesized by a facile hydrothermal method using two different source materials of Ti: tetrabutyl titanate (TBT) and commercial TiO_2_ powder (P25). For respective series with the same source materials, we investigated additions that optimized the nonlinear optical properties (NLO) and optical limiting (OL) performances, and we explored the relationships between structural diversity and performance. Several characterization techniques, including X-ray powder diffraction (XRD), X-ray photoelectron spectroscopy (XPS), transmission electron microscopy (TEM), Fourier-transform infrared spectroscopy (FTIR), Raman spectroscopy, and diffuse reflectance ultraviolet-visible spectroscopy (UV-Vis) were conducted to confirm the microstructures and chemical states of as-prepared materials. This indicated the existence of the Ti–O–C bond between rGO sheets and TiO_2_ particles and the reduction from precursor graphene oxide (GO) to rGO. The results of UV-Vis spectra revealed that the TiO_2_/rGO nanocomposites showed smaller band gaps than bare TiO_2_. A nanosecond open-aperture Z-scan technique at 1064 nm was applied to investigate NLO and OL properties. TiO_2_/rGO nanocomposites exhibited enhanced NLO and OL performances, arising from synergistic effects, compared to individual components. The TBT series samples performed better than the P25 series, presumably relevant to dimensional effects.

## 1. Introduction

Nowadays, because of their good monochromaticity, collimation, high brightness, and good coherence, lasers have been widely used in many respects of our lives. Optical lens, internal optical components, sensors, etc., in optoelectronic devices are too vulnerable to sustain strong laser radiation attacks because of their high cumulative optical gain, which results in the material failing to recover and a loss of function. The demand for laser protection for low-damage threshold and high-sensitivity optical sensors, such as fragile human eyes and optical apparatuses that are easily destroyed, has attracted a lot of attention. Optical limiting materials are capable of exhibiting significant promise for laser protection because of their nonlinear optical (NLO) properties [1,2,3,4]. Remarkable nonlinear optical properties have been reported in numerous materials, which mainly comprise organic compounds, inorganic semiconductors, noble metal clusters, fullerenes, graphene, and carbon nanotubes (CNTs) [5,6,7,8,9,10,11,12,13,14].

In comparison with graphene, there are some remaining oxygen-containing groups in reduced graphene oxide (rGO) sheets. The existence of remaining groups can ensure attachment between rGO and other optical limiting (OL) materials [15,16]. Compared to graphene oxide (GO), reduced graphene oxide (rGO) partially renovates the π-electron conjugation and shows stronger nonlinear optical properties and optical limiting performances. In addition, rGO provides better electrical conductivity and a larger size π-conjugated system, on average [17,18]. Lim et al. [19] investigated graphenes, which were spread out in varied dispersants, and found they could exhibit broadband nonlinear absorption as a result of an intense matrix effect, for which there was remarkable optical limiting clamp threshold of 10 mJ/cm^2^. The research of Kavitha et al. [20] found that reduced graphene oxide–zinc oxide nanohybrids exhibited OL enhancements compared to graphene oxide (GO) and ZnO.

Titanium dioxide (TiO_2_) has been widely reported as a semiconductor material with excellent photoelectric properties. It has been investigated in the field of photocatalysis because of its extraordinary optical properties and chemical stability [21,22]. However, single-component materials are less competitive in many properties than multicomponent composites. Nanocomposite materials containing TiO_2_ are more attractive than bare TiO_2_ [23,24,25]. In addition, TiO_2_ with different particle sizes has shown some interesting properties [26,27,28]. In light of the similarity between the mechanisms of photocatalysis and that of optical limiting, we can get inspiration from the idea of photocatalysis. Since most investigations focus on the photocatalysis aspect, there are few reports concerning the nonlinear optical properties and optical limiting properties of TiO_2_-based nanohybrids.

Knowing that particle sizes of TiO_2_ will cause differences in photocatalytic H_2_-production activity [27], in this study we investigated the effects of different TiO_2_ particle sizes of TiO_2_/rGO nanocomposites on nonlinear optical (NLO) properties and optical limiting (OL) performances. TiO_2_/rGO nanocomposites with different TiO_2_ particle sizes were obtained using varied Ti source materials: tetrabutyl titanate (TBT) and P25. As two series of source materials of Ti, the experiments were carried out in parallel. For respective series with the same source materials, we investigated the additions that optimized NLO and OL performances, in addition to characterization, to confirm the structures of the resultant materials.

## 2. Materials and Methods

### 2.1. Reagents

Purified natural graphite powder with an average particle size less than 48 μm was purchased from Nanjing XFNANO Materials Tech Co., Ltd (Nanjing, China). Ninety-eight percent sulfuric acid (H_2_SO_4_), 38% hydrochloric acid (HCl), 30% hydrogen peroxide (H_2_O_2_), absolute ethanol (C_2_H_5_OH), and sodium nitrate (NaNO_3_) were purchased from Sinopharm Chemical Reagent Co., Ltd. (Shanghai, China) Potassium permanganate (KMnO_4_), tetrabutyl titanate (C_16_H_36_O_4_Ti), P25 (anatase, hydrophilic, and lipophilic grades), and *N*-Methyl pyrrolidone (C_5_H_9_NO) were purchased from Aladdin (Shanghai, China). All chemicals were analytically pure reagents, and the water used in the experiments was deionized water. For the sake of brevity, TBT denotes tetrabutyl titanate and NMP denotes *N*-Methyl pyrrolidone.

### 2.2. Instruments and Measurements

X-ray powder diffraction (XRD) experiments were conducted using a D2 PHASER diffractometer (Bruker AXS, Billerica, MA, USA) under Cu-Ka radiation (k = 0.1542 nm). Transmission electron microscopy (TEM) studies were analyzed on a transmission electron microscope (JEM-2100, JEOL, Tokyo, Japan) with an operating voltage of 200 kV. X-ray photoelectron spectroscopy (XPS) was characterized on a Thermo Scientific™ ESCALAB™ Xi^+^ X-ray Photoelectron Spectrometer (XPS) microprobe with an excitation source of Al Kα at 150 eV. Fourier-transform infrared (FTIR) spectra were carried out on a Nicolet^TM^ iN10^TM^ MX Infrared Imaging Microscope (Thermo Scientific, Waltham, MA, USA) using spectroscopic-grade KBr pellets for measurements ranging from 4000 cm^−1^ to 400 cm^−1^. Raman spectroscopy was carried out on a DXR Raman Microscope (Thermo Scientific, Waltham, MA, USA) with a working excitation wavelength of 532 nm. UV-Vis spectroscopy was recorded on a HITACHI (Tokyo, Japan) U-4100 UV-VIS-NIR Spectrophotometer ranging from 200 to 800 nm. The above experiments were carried out at room temperature, except for TEM.

Nonlinear optical properties of the samples were analyzed on an open-aperture Z-scan device using 6 ns laser pulses at 1064 nm generated by a Q-switched Nd:YAG laser. A beam splitter typically splits a laser beam into two beams, one of which hits the test sample and one of which acts as the incident light. A convex lens with a focal length of 15 cm focused the incident laser beam in the Z direction. All samples were dispersed in *N*-Methyl pyrrolidone (NMP) and placed in 10 mm-thick quartz cuvettes for the Z-scan test with a pulse repetition rate of 10 Hz. During the test process, the samples were set on a on a sample stage that was computer controlled to move the sample along the Z axis relative to the focus. To estimate their NLO properties and OL performances, the suspensions were prepared with uniform linear transmittances of 60% at 1064 nm.

### 2.3. Synthesis of Graphene Oxide (GO)

Graphene oxide (GO) was obtained by a modified method of Hummers and Offeman. Graphite powder (2 g), sodium nitrate (1 g), and 98% sulfuric acid (100 mL) were mixed into a 500 mL beaker that had been cooled to 0 °C with an ice bath. The ingredients were stirred for 30 min while the temperature was controlled below 10 °C. Then, potassium permanganate (6 g) was divided into four identical batches and gradually added to the mixture every 15 min. After that, the mixture was heated to 35 °C and mixed around at 35 °C for 2 h. Slowly and gently, 100 mL of deionized water was decanted to the mixture within 30 min, and then the mixture was heated up to 95 °C. The reaction mixture was stirred at 95 °C for 10 min, and 200 mL distilled water was added slowly. The suspension was treated with several drops of 30% hydrogen peroxide until the color of the suspension was changed from brown to luminous yellow, which manifested the generation of grapheme oxide. The dispersion was washed with 10% HCl solution and deionized water using a centrifuge and decantation until the pH of the suspension was about 7. A portion of the product was treated by vacuum freeze-drying, and the other product was preserved as aqueous solution in a reagent bottle.

### 2.4. Synthesis of Tetrabutyl Titanate (TBT)-TiO_2_/rGO Nanocomposites

GO (50 mg) was first dissolved in deionized water (45 mL), and the solution was ultrasonically treated for 90 min. Then, absolute ethanol (15 mL) was decanted to the solution, and the blend was ultrasonically treated for a further 30 min to form a homogeneous solution. After that, a certain amount of TBT was dropped into the blend to form a suspension, which was magnetically stirred for 30 min. Next, the suspension was ultrasonically treated for 30 min. Same as the former procedures, the suspension was stirred for 30 min and was ultrasonically treated for 30 min. All treatments contributed to a uniform suspension. Finally, the suspension was decanted into a 90 mL hydrothermal synthesis reactor, and the reactor was under heat preservation at 150 °C for 5 h. The resultant product was collected by suction filtration. It was washed first with distilled water, followed by C_2_H_5_OH for a few times, and then desiccated at 40 °C for several hours. In order to determine the influence of TiO_2_ content on the NLO properties and OL performances of the TiO_2_/rGO nanocomposites, nanocomposites with a series of different TiO_2_ contents were prepared by adding corresponding different amounts of TBT, which served as a source of TiO_2_ in the TiO_2_/rGO nanocomposites. The weights of TBT added in the experiment were diverse (0.10, 0.20, 0.25, 0.30, 0.35, and 0.40 g), and the corresponding product was recorded as *x*g TBT, where *x* = 0.10, 0.20, 0.25, 0.30, 0.35, or 0.40, respectively. For comparison, pure TiO_2_ was synthesized by the same hydrothermal condition without the addition of GO. All prepared samples of TBT series are shown in Table 1.

### 2.5. Synthesis of P25-TiO_2_/rGO Nanocomposites

To determine the effect of different particle sizes of TiO_2_ on the microstructure and OL properties of the TiO_2_/rGO composites, P25 (relative to TBT) was selected as another base for the TiO_2_/rGO composites. During the above synthesis procedures of TiO_2_/rGO nanocomposites, TBT was replaced by P25 to repeat the same experiment process. Also, the weights of P25 added to the reaction system were different (0.05, 0.10, 0.25, 0.40, or 0.75 g), and the corresponding product was recorded as *x*g P25, where *x* = 0.05, 0.10, 0.25, 0.40, or 0.75, respectively. All prepared samples of P25 series are shown in Table 2.

## 3. Results and Discussion

### 3.1. Characterization of TiO_2_/rGO Nanocomposites

#### 3.1.1. X-ray Powder Diffraction (XRD) Studies

**Figure 1:**
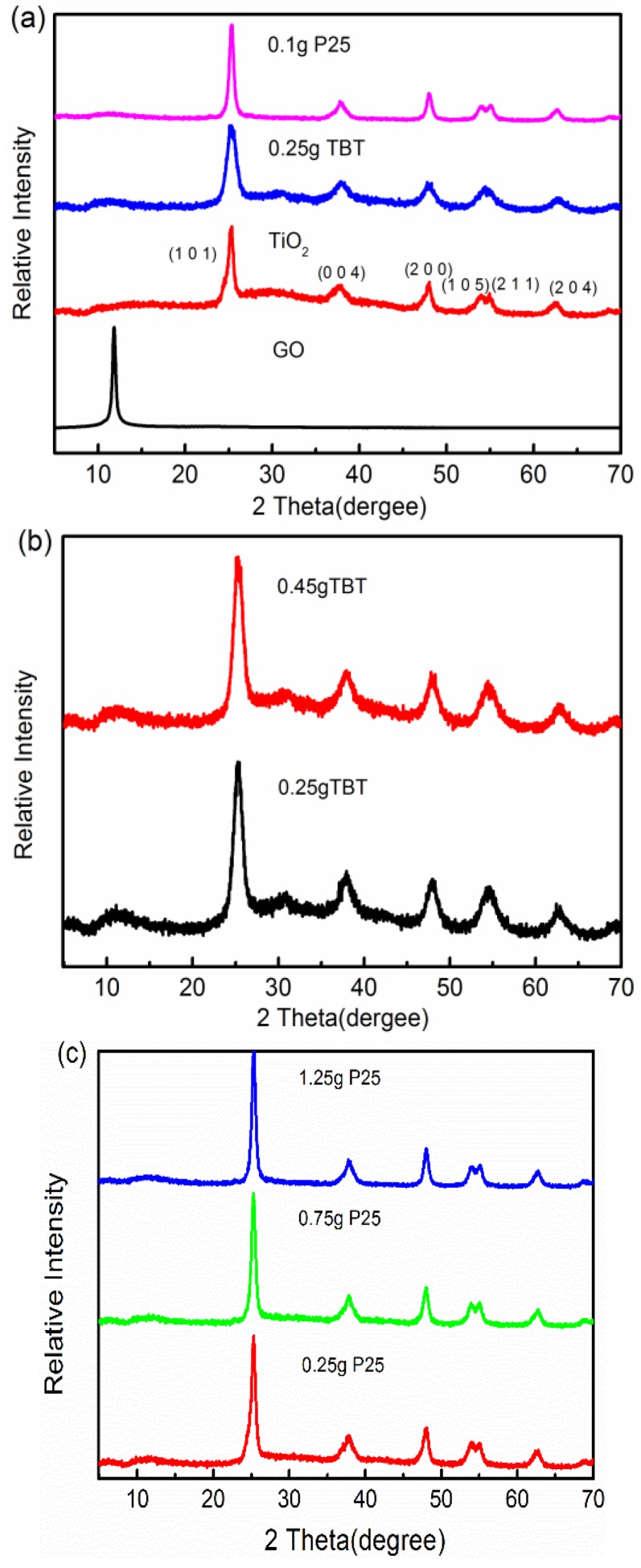
(**a**)X-ray powder diffraction (XRD) patterns of 0.1 g P25, 0.25 g TBT, TiO_2_, and graphene oxide (GO); (**b**) XRD patterns of the weights of different TBT added in TiO_2_/rGO nanocomposites; and (**c**) XRD patterns of the weights of different P25 added in TiO_2_/rGO nanocomposites.

The XRD patterns of precursor GO, bare TiO_2_, and two series of TiO_2_/rGO nanocomposites with different sources of Ti are presented in Figure 1. There was a strong sharp peak at 2θ = 11.8° shown in the patterns, which indicated a large interlayer spacing of 0.75 nm according to Bragg’s Law. The layer spacing was larger than that of graphene, which was 0.375 nm, and the increase was caused by the existence of a large number of oxygen-containing functional groups on the surface of the GO sheets. Compared to Joint Committee on Powder Diffraction Standards (JCPDS) Card No. 21-1272, all XRD patterns of the two series of TiO_2_/rGO nanocomposites and bare TiO_2_ could be indexed to anatase, which indicated the spontaneous product of TiO_2_ during the hydrothermal process. Moreover, it displayed no characteristic diffraction peaks related to rutiles and brookites in Figure 1, which indicated the TiO_2_ was free of rutiles and brookites [29,30]. Although the Ti-source materials of the two samples were different, 0.1 g P25 and 0.25 g TBT samples exhibited similar patterns from Figure 1a. Likewise, the TiO_2_/rGO nanocomposites with different amounts of TBT exhibited similar XRD patterns (Figure 1b). The same phenomenon were also observed in Figure 1c, which revealed that the lattice structures of both TiO_2_ and rGO were not affected. The addition amounts of source materials had no bearing on the XRD patterns of the TiO_2_/rGO nanocomposites either. The small peaks around 11°, shown in Figure 1b, were indexed to the slightly redundant GO and the baseline drift of TiO_2_ [31]. Notably, no apparent peaks corresponding to rGO were observed in the XRD patterns of TiO_2_/rGO nanocomposites, probably because the characteristic peak of rGO at about 25° was covered by the characteristic peak of anatase at 25.3° [32].

#### 3.1.2. X-ray Photoelectron Spectroscopy (XPS) Spectra Analysis

**Figure 2:**
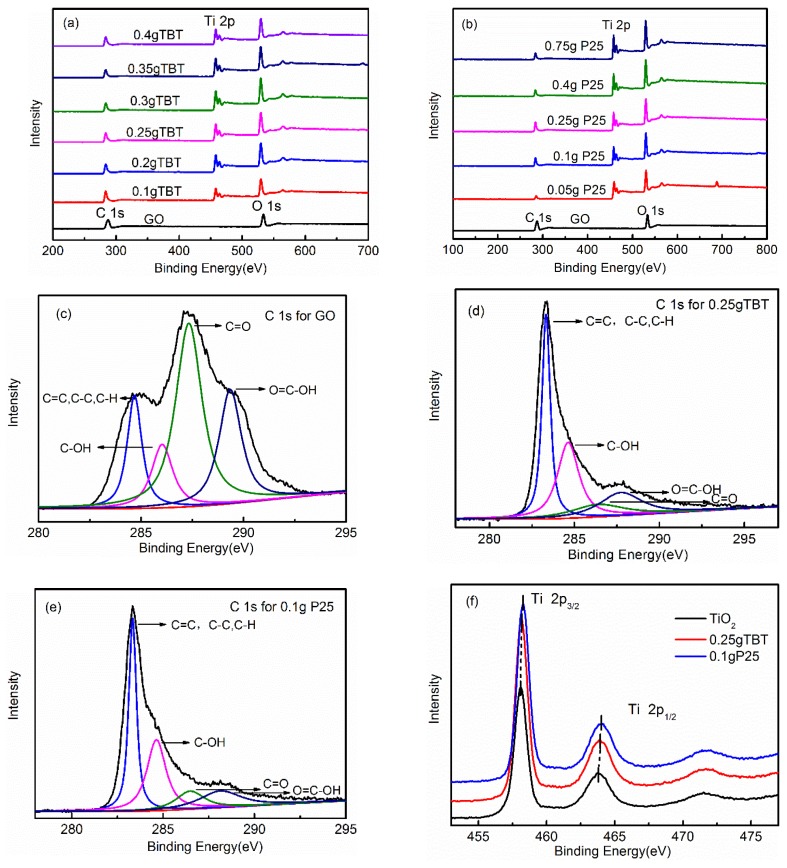
(**a**) X-ray photoelectron spectroscopy (XPS) spectra of GO, 0.1 g TBT, 0.2 g TBT, 0.25 g TBT, 0.3 g TBT, 0.35 g TBT, and 0.4 g TBT. (**b**) XPS spectra of GO, 0.05 g P25, 0.1 g P25, 0.25 g P25, 0.4 g P25, and 0.75 g P25. C 1s core-level spectra for (**c**) GO, (**d**) 0.25 g TBT, (**e**) 0.05 g P25, and (**f**) Ti 2p core.

GO, TiO_2_, and TiO_2_/rGO nanocomposites were characterized by XPS spectra analyses to confirm varied chemical states. Figure 2a,b displays XPS spectra of a series of samples with different amounts of TBT and P25. Compared with the spectrum of GO, there was a new peak assigned to Ti 2p in spectrum of every TiO_2_/rGO nanocomposite sample. Figure 2f shows the Ti 2p core-level spectra of TiO_2_, the 0.25 g TBT sample, and the 0.1 g P25 sample. The two typical peaks in this region were associated, respectively, with the Ti 2p_1/2_ and Ti 2p_3/2_ splitting photoelectrons of the Ti^4+^ chemical state [33]. For the 0.25 g TBT sample, the two peaks were centered at 463.93 and 458.18 eV, while those of GO were at 463.78 and 458.08 eV. For the 0.1 g P25 sample, the two peaks were centered at 464.03 and 458.28 eV. The gap in GO between the two Ti-bands was about 5.7 eV, while the gaps in the 0.25 g TBT sample and the 0.1 g P25 sample were both 5.75 eV [34]. The fine distinction in the position of the two peaks may correspond to the interplay between TiO_2_ and the rGO, which probably indicates the existence of Ti–O–C bonds in the TiO_2_/rGO nanocompsites. To study the carbon chemical state in the TiO_2_/rGO nanocompsites, C 1s XPS core-level spectra of GO, the 0.25 g TBT sample, and the 0.1 g P25 sample were shown in Figure 2c–e. The deconvoluted peaks of GO were centered at 284.68, 286.03, 287.33, and 289.53 eV, respectively. The C=C, C–C, and C–H bonds, which contained no oxygen groups, usually corresponded to the peak centered at 284.68 eV, while the peaks at 286.03, 287.33, and 289.53 eV were attributed to the hydroxyl (C–OH), carbonyl (C=O), and carboxyl (O=C–OH) groups, respectively [34,35,36,37,38]. The 0.25 g TBT sample showed different deconvoluted peaks at 283.33, 284.62, 286.38, and 287.68 eV, respectively. According to the results of deconvoluted peaks, the intensities of peaks associated with the C=C, C–C, and C–H bonds were sharply enhanced after the hydrothermal process, while the intensities of peaks ascribed to the C=O and O=C–OH bonds decreased dramatically. Figure 2e shows the similar results of the 0.1 g P25 sample. The changes about the intensity of the peaks implied the reduction of GO as it transformed to rGO during the hydrothermal treatment. There was no peak related to Ti–C bonds at about 282 eV [39] in the TiO_2_/rGO samples, which meant there was no carbon doped into the lattice of TiO_2_.

#### 3.1.3. Transmission Electron Microscopy (TEM) Studies

**Figure 3:**
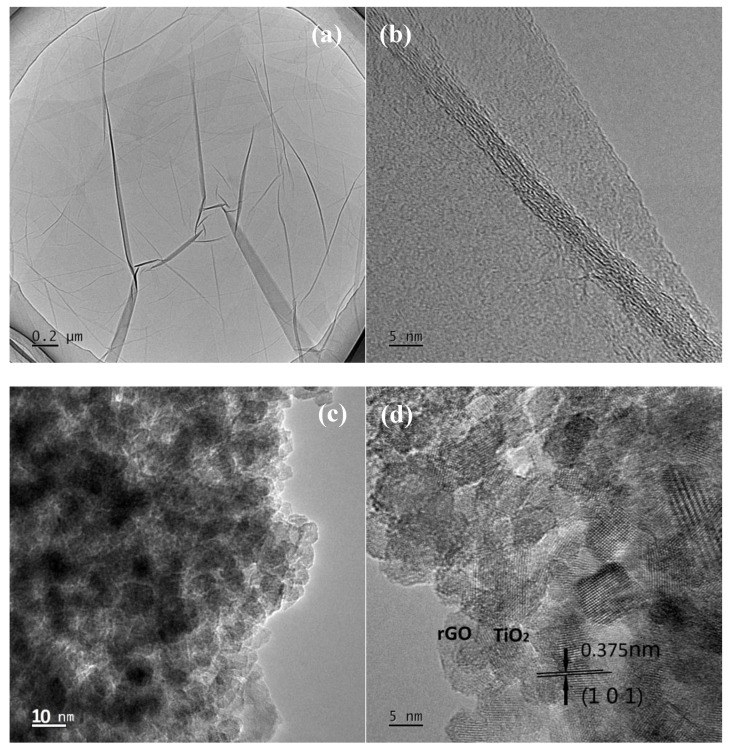

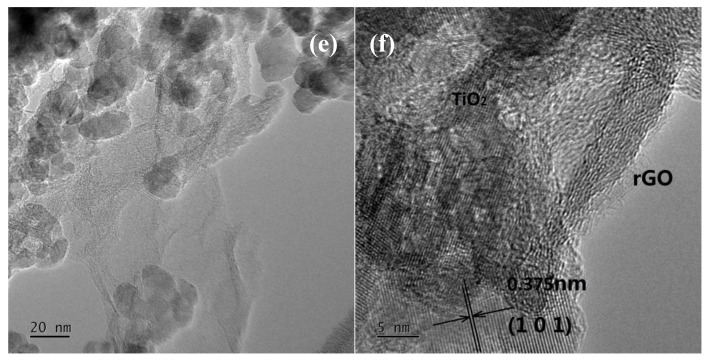
Transmission electron microscopy (TEM) images of GO (**a**), 0.25 g TBT (**c**), and 0.1 g P25 (**e**); High-resolution TEM (HRTEM) images of GO (**b**), 0.25 g TBT (**d**), and 0.1 g P25 (**f**).

Figure 3a,c,e shows the transmission electron microscopy (TEM) images and Figure 3b,d,f shows high-resolution TEM (HRTEM) images of GO, the 0.25 g TBT sample, and the 0.1 g P25 sample. Figure 3a,b displays that GO, one of the precursors of the nanocomposites, consisted of single- and multilayer GO sheets. Since the GO sample was dried and then dispersed for TEM testing, the degree of agglomeration was acceptable. Figure 3c,d displays the TEM and HRTEM images of the 0.25 g TBT sample, while Figure 3e,f shows those of the 0.1 g P25 sample. According to Figure 3c,e, the TiO_2_ particle size of the 0.25 g TBT sample was about 5 nm, while that of the 0.1 g P25 was about 20 nm. In that case, the TBT series samples had larger specific surface areas than the P25 series, which indicated that the contact area between TiO_2_ and rGO of the TBT series samples was larger than that of the P25 series. Therefore, the electron transfer process performed better in the TBT series, which was conducive to the separation of photogenerated electrons and holes and improved the optical limiting performances. HRTEM images displayed the distinct lattice fringes. According to Figure 3d,f, the interplanar spacing of TiO_2_ particles could be measured and calculated as 0.375 nm, which was indexed to the (1 0 1) plane of TiO_2_ with an anatase structure. Apparently, rGO sheets were almost covered by anchored TiO_2_ nanoparticles, providing the possibility of efficient electronic conducting.

#### 3.1.4. Fourier-Transform Infrared (FTIR) Spectra Analysis

**Figure 4:**
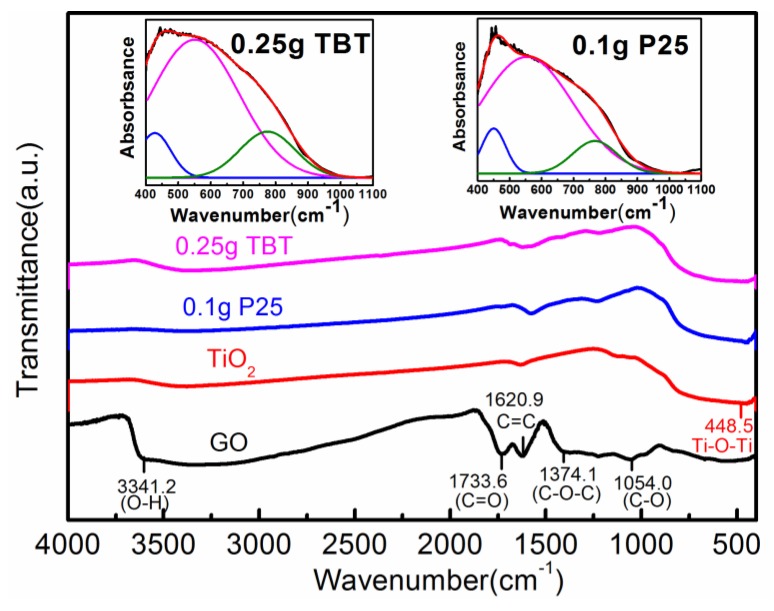
Fourier-transform infrared (FTIR) spectra of GO, TiO_2_, 0.25 g TBT sample, and 0.1 g P25 sample. The inset shows the deconvolution results of the partial spectra of 0.25 g TBT and 0.1 g P25.

For the purpose of confirming the bonding characteristics of functional groups in the as−prepared samples, Fourier-transform infrared spectroscopy (FTIR) was utilized. Figure 4 displays the FTIR spectra of GO, TiO_2_, the 0.25 g TBT sample, and the 0.1 g P25 sample. Several strong absorption bands of oxygen-containing groups were shown in the FTIR spectrum of GO. The typical peaks at 3341, 1733, 1620, 1374, and 1054 cm^−1^ corresponded to the stretching vibration of hydroxyl (O–H), the stretching vibration of carboxy/carbonyl (C=O), the skeletal stretching vibration of aromatic (C=C), the stretching vibration of epoxy/ether (C–O–C), and the stretching vibration of alkoxy/alkoxide (C–O) [40,41], respectively. The spectra of 0.25 g TBT and 0.1 g P25 indicated that the intensities of the bands associated with the oxygen-containing functional groups decreased markedly, implying that GO was reduced to rGO. The ratiocination was consistent with the result of the deconvoluted XPS spectra. Nevertheless, there was a small amount of oxygen-containing functional groups attached to rGO sheets. The broad band absorption between 448 and 1000 cm^−1^ can be considered as the stretching vibration of Ti–O–Ti bonds combined with the stretching vibration of Ti–O–C bonds [30,40,42,43,44]. The inset of Figure 4 shows the deconvoluted FTIR spectra ranging from 1100 to 400 cm^−1^. For the 0.25 g TBT sample, the band at 775 cm^−1^ was ascribed to the stretching vibration of the Ti–O–C bonds. The other two bands at 427 and 551 cm^−1^ were assigned to the stretching vibration of Ti–O–Ti bonds [43]. For the 0.1 g P25 sample, the corresponding bands were located at 767, 450, and 553 cm^−1^. The existence of the Ti–O–C bands demonstrated that the interaction between the oxygen-containing functional groups of GO and TiO_2_ were strong enough to keep the two components attached tightly during the hydrothermal process, and ultimately the Ti–O–C bonds were generated.

#### 3.1.5. Raman Spectra Analysis

**Figure 5:**
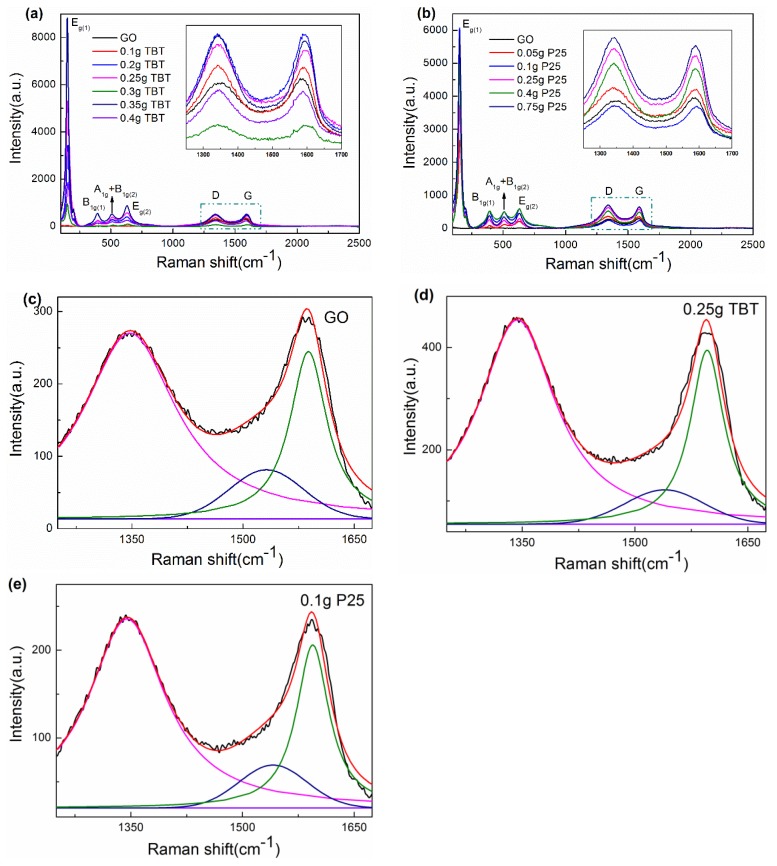
Raman spectra of GO, 0.25 g TBT sample (**a**) and 0.1 g P25 sample (**b**). The inset shows the D-band and G-band partial Raman spectra ranging from 1250 to 1700 cm^−1^. Raman spectra of GO (**c**), 0.25 g TBT sample (**d**), and 0.1 g P25 sample (**e**) with the corresponding curve fitted bands.

Raman spectroscopy can serve as one type of technological means to acquire overall microstructure information of GO and two series of TiO_2_/rGO nanocompsites. Typical sharp peaks are associated with different types of vibration modes of the anatase lattice. Both TBT and P25 samples exhibited similar characteristic bands at about 152, 390, 506, and 630 cm^−1^, which contributed to E_g(1)_, B_1g(1)_, A_1g_ + B_1g(2)_, and E_g(2)_ modes of anatase, respectively [45,46,47,48]. Usually, peaks associated with Eg mode were strongly affected by the particle size. As the particle size increased, the half-width at half maximum (HWHM) of the peak increased, and the peak blue-shifted [26,28,49]. But these influences decreased when the particle size was big enough (>20 nm) to lose the quantum size effect. The other characteristic bands at 1343 and 1589 cm^−1^ were associated with the D-band and G-band of graphitization, respectively. G-band was associated with the in-plane vibration of sp^2^ hybridized carbon atoms [50], while D-band revealed disordered vibrations of carbon atoms. The intensity ratio of D-band to G-band (I_D_/I_G_) offered a measure of the defects/degree of order in rGO. The I_D_/I_G_ for GO, 0.05 g P25, 0.1 g P25, 0.25 g P25, 0.4 g P25, and 0.75 g P25 samples were 0.93467, 1.03598, 1.0217, 1.08191, 1.07916, and 1.07973, respectively. The intensity of ratios of 0.1 g TBT, 0.2 g TBT, 0.25 g TBT, 0.3 g TBT, 0.35 g TBT, and 0.4 g TBT were 1.02919, 1.00269, 1.0735, 1.06654, 1.0591, and 1.02956, respectively. Compared to that of GO, increased intensity ratios of I_D_/I_G_ were observed for TiO_2_/rGO composites. The increment indicated a smaller average size of the in-plane sp^2^ domains and fragment process of GO during the hydrothermal reaction [27]. Figure 5c–e shows the curve fitting for GO, the 0.25 g TBT sample, and 0.1 g P25 sample, respectively. Lorentzian fitting was applied to analyze peaks at around 1350 and 1590 cm^−1^, while Gaussian fitting was applied to analyze the peak at around 1520 cm^−1^. The two Lorentzian lines were assigned to the aforementioned D-band and G-band vibrations, as the Gaussian curve corresponded to the amorphous graphitic phase [50]. We calculated the intensity ratio of the Gaussian curve versus D-band Lorentzian curve as a measure of the content of the amorphous graphitic phase. The ratios were 0.3, 0.26, and 0.28 for GO, the 0.25 g TBT sample, and the 0.1 g P25 sample, respectively. The decreased ratio indicated that the 0.25 g TBT sample displayed a higher degree of order.

#### 3.1.6. UV-Vis Absorption Spectra Analysis

**Figure 6:**
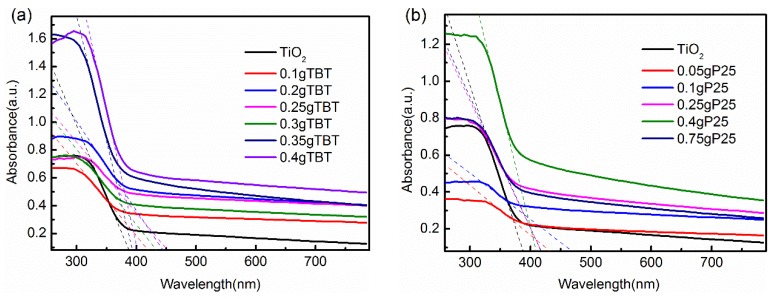
UV-Vis absorption spectra of P25 series samples (**a**) and TBT series samples (**b**).

Figure 6 displays the UV-Vis absorption spectra of TiO_2_, TBT samples (a) and P25 samples (b). Bare TiO_2_ showed a characteristic absorption edge at about 390 nm, which was associated with a charge transfer from the valence band to the conduction band [29]. Absorption wavelength threshold (λ_g_) and corresponding band gap (E_g_) were calculated and shown in Table 3 according to reference [51]. Compared to bare TiO_2_, all TiO_2_/rGO nanocomposites, whether TBT series samples or P25 series samples, exhibited enhanced light absorption in the visible region and a smaller band gap. Moreover, the absorption edge of TiO_2_/rGO nanocomposites showed different degrees of red shift, which probably was due to the decreased band gap of TiO_2_ and the oxygen vacancies existing on the surface of TiO_2_ [29,30,52,53,54], corresponding to the results of FTIR spectra and XPS spectra. The decreased band gap indicated a larger possibility for photon-generated carriers to transfer, which increased the kinetic energy of the carriers to disperse the laser energy.

### 3.2. Characterization of Nonlinear Optical (NLO) and Optical Limiting (OL) Performaces

Optical limiting behavior was assessed using the Z-Scan technique, which was used to characterize the OL performances. During the test process, the detectors collected and recorded the incident laser energy and the transmitted laser energy, and transmittance was obtained when the sample was translated through the focal plane of a tightly focused beam. The transmittance of samples can be regarded as a function of the displacement distance (Z) [11]. When the material was translated close to the focus of the beam, the intensity of the incident beam increased, and the materials exhibited optical limiting effects as a result of its nonlinear optical properties, such as the decline of the transmittance. It was the internal mechanism determined by the material structure that caused a decrease in the transmittance. The optical limiting mechanism of TiO_2_/rGO nanocompsites probably included reverse-saturated absorption (RSA), two-photon absorption (TPA), nonlinear scattering (NLS), or a combination of these mechanisms [47,55]. Nonlinear performances were regarded as better when the transmittance decreased more than that of the lower transmittance.

**Figure 7:**
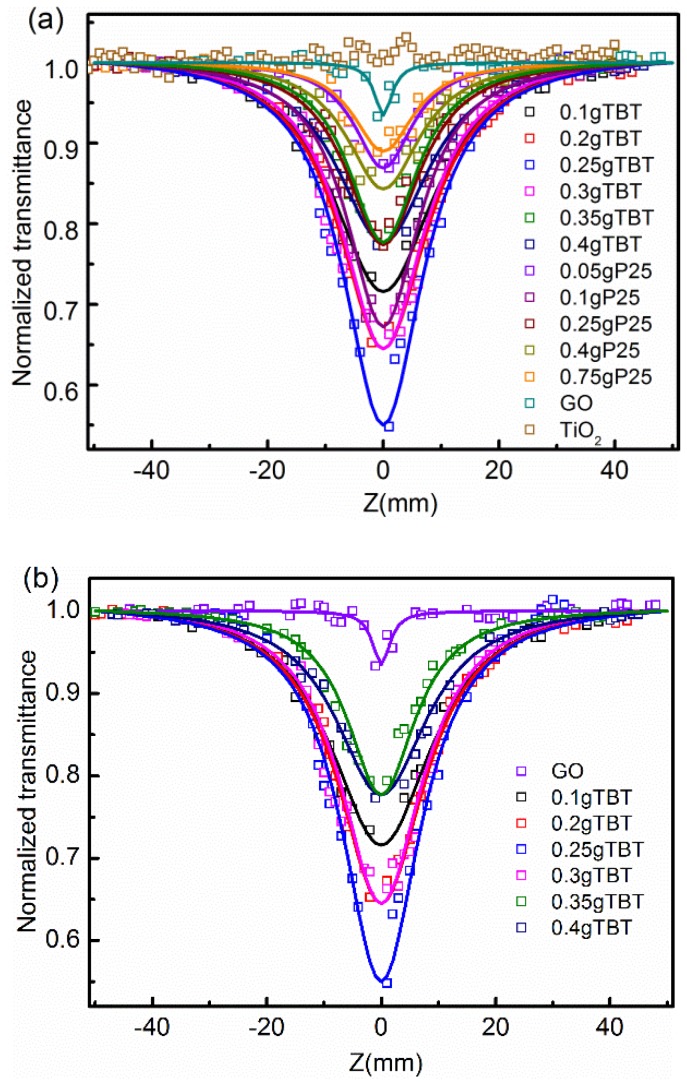

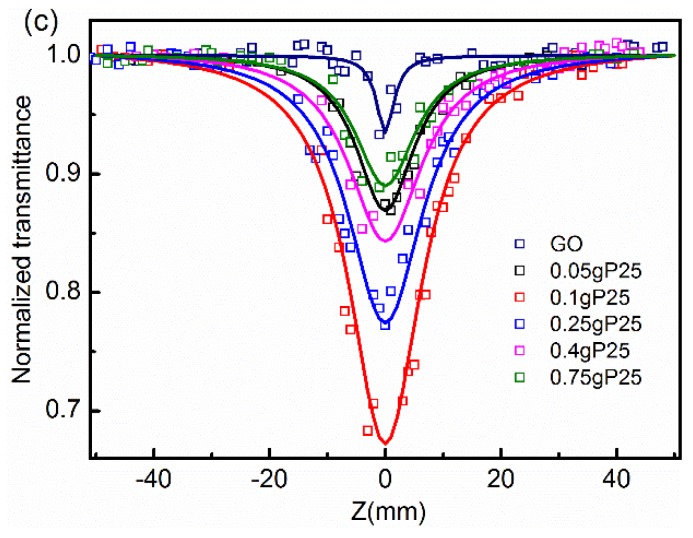
Open aperture Z-scan normalized transmittance curves using 6 ns laser pulses at 1064 nm for GO, TiO_2_, TBT samples, and P25 samples (**a**), GO and TBT series samples (**b**), GO and P25 series samples (**c**).

Figure 7 displays the open aperture Z-scan results of the as-prepared materials at 300 μJ. It was apparent that almost all samples showed only a valley at the focus, while TiO_2_ showed slight nonlinearity. As previously mentioned, a decrease of normalized transmittance indicated the NLO performances. The 0.25 g TBT sample showed the best nonlinear optical performances, from Figure 7a, which performed better than the single-walled carbon nanotubes reported in reference [56]. For TBT series, the 0.25 g TBT sample showed the best nonlinear optical performance, while the 0.1 g P25 sample performed best in the P25 series. As for the variation tendency of the respective series, the optical limiting performances increased first and then decreased with the increase of the addition of Ti precursors. Here, results of the TEM were easily combined. When the amount of Ti precursors was less than the optimal addition, rGO sheets were capable of accepting more photo-induced electrons and were not “saturated”. While the amount of Ti precursors exceeded the optimal addition, the interaction between TiO_2_ particles was greater than that of TiO_2_ particles and rGO, resulting in restrictions on the charge transfer process. Also, the gap between the OL performances of 0.25 g TBT and 0.1 g P25 was observed. As a matter of fact, the gap between two samples, to a great extent, contributed to the differences in the microstructures of the two series samples. Raman spectra indicated that TBT series samples had a better degree of order that was conducive to charge transfer. TEM displayed that TBT samples had smaller particle sizes and larger specific surface areas. The specific surface area was an important parameter in the performance measurement for the nanoparticles. To a certain extent, the quantum size effect in TBT samples played a synergistic role in OL performances.

For further quantitative analysis, the Crank–Nicolson finite-difference scheme was used to calculate the nonlinear absorption coefficient β [57]. The values of β for GO, 0.25 g TBT sample, and 0.1 g P25 sample were 0.035, 1.39, and 0.87 cm/GW, respectively. It showed that β for 0.25 g TBT was 39 times larger than that of GO, and β for 0.1 g P25 was 24 times larger than that of GO. The result confirmed that the TiO_2_ addition to GO enhanced nonlinear absorption [11]. Based on the above results, the TiO_2_/rGO composites exhibited better optical limiting performances than their individual components, probably corresponding to the reduction from GO to rGO and synergetic effects. Compared to GO, the TiO_2_/rGO composites displayed larger π-electron conjugated systems and degrees of order, which revealed better NLO and OL performances [19], corresponding to the aforementioned XPS and Raman results. The 0.25 g TBT sample and 0.1 g P25 sample were the best examples of making perfect use of synergetic effects in their respective series [13,14]. Because of the strong interplay between TiO_2_ and rGO, photo-induced electrons can be transferred to rGO sheets instantly as the TiO_2_ particles absorbed laser energy [31,58]. The main mechanisms resulting in the nonlinear optical properties were reverse-saturated absorption (RSA), free carrier absorption (FCA), and nonlinear scattering (NLS) [24,43]. In addition, energy can be easily transferred into the solvent. Local overheating might cause microbubbles in the solvent, which were capable of showing strong nonlinear scattering effects [59]. In terms of the RSA and NLS mechanisms, the sample with a larger specific surface area had a bigger contact area with the laser and solvent, corresponding to stronger reverse-saturated absorption and nonlinear scattering. In addition, TiO_2_ particle size impacted the low-frequency E_g_ mode [26], which was probably relative to the optical properties. Compared to the 20 nm of P25 samples, the 5 nm TiO_2_ particle size of TBT samples exhibited better NLO and OL properties.

## 4. Conclusions

A facile hydrothermal method was adopted in this research to synthesize TiO_2_/reduced graphene oxide (rGO) nanocomposites with TBT and P25 as different Ti-sources. A combination of characterization methods verified that the TiO_2_ particles and rGO sheets were attached by Ti–O–C bonds. The NLO and OL performances of the TiO_2_/rGO nanocomposites were measured using the Z-scan technique of 6 ns laser pulses at 1064 nm. The 0.25 g TBT sample and 0.1 g P25 sample showed prominent NLO and OL performances in their respective series prior to the individual components. Significant enhancement corresponded to the synergistic effect between TiO_2_ particles and rGO sheets. The 0.25 g TBT sample performed better than 0.1 g P25 in OL properties. The TiO_2_ particle size of the 0.25 g TBT sample was about 5 nm, and that of the 0.1 g P25 sample was about 20 nm. Dimensional differences may account for the distinct performances.

## Figures and Tables

**Table 1:** The prepared samples of the tetrabutyl titanate (TBT) series.

Samples	Source Materials of TiO_2_	Weight of Source Materials
0.1 g TBT	TBT	0.10 g
0.2 g TBT	TBT	0.20 g
0.25 g TBT	TBT	0.25 g
0.3 g TBT	TBT	0.30 g
0.35 g TBT	TBT	0.35 g
0.4 g TBT	TBT	0.40 g

**Table 2:** The prepared samples of the P25 series.

Samples	Source Materials of TiO_2_	Weight of Source Materials
0.05 g P25	P25	0.05 g
0.1 g P25	P25	0.10 g
0.25 g P25	P25	0.25 g
0.4 g P25	P25	0.40 g
0.75 g P25	P25	0.75 g

**Table 3:** The Absorption wavelength thresholds and corresponding band gaps of all samples.

TBT Series Sample			P25 Series Samples		
Samples	λ_g_ (nm)	E_g_ (eV)	Samples	λ_g_ (nm)	E_g_ (eV)
0.1 g TBT	435	2.85	TiO_2_	396	3.13
0.2 g TBT	457	2.71	0.05 g P25	462	2.68
0.25 g TBT	468	2.65	0.1 g P25	501	2.48
0.3 g TBT	449	2.76	0.25 g P25	429	2.89
0.35 g TBT	399	3.11	0.4 g P25	416	2.98
0.4 g TBT	407	3.05	0.75 g P25	428	2.90
